# Be mindful of potential pitfalls when using the Cre-LoxP system in cancer research

**DOI:** 10.18632/oncoscience.591

**Published:** 2023-11-14

**Authors:** Piotr Czarnota, Jaroslaw Cisowski

**Keywords:** LoxP, dedifferentiation, transdifferentiation, epithelial-to-mesenchymal, cancer

The Cre-LoxP system is widely used to conditionally modify gene expression in mouse models of cancer and other diseases. It is based on specific recognition and cutting of LoxP elements embedded in the genome by Cre recombinase [1]. The genetic modifications induced by Cre-LoxP can be spatially and/or temporarily restricted to specific tissues due to the use of cell-specific and/or inducible gene promoters driving *Cre* expression. However, the specificity of *Cre* expression depends on the cell type fidelity of these promoters. Thus, the appropriate interpretation of experimental results involving the Cre-LoxP system requires knowledge of the activity pattern of a given gene promoter in organs and tissues. In this regard, it is worth emphasizing that the activities of some gene promoters utilized to drive Cre expression are not specific to intended cell types. As an example, many of the pancreatic endocrine and ductal cell-specific promoters are also expressed in some brain neurons, liver, stomach, and intestines, and may be temporarily active at early stages of development [2], resulting in the lack of specificity of genetic recombination. This problem is not limited by any means to the pancreas, e.g., a *Lys2* promoter, a gene encoding a protease Lysozyme M, widely used to delete genes in the myeloid lineage, is also active in type 2 pneumocytes in the lungs [3]. Moreover, microvesicles-mediated transfer of Cre mRNA into neighboring cells may also contribute to unfaithful labeling of cells and lead to false interpretation of results [4].

The use of Cre-LoxP system to interrogate cancer biology poses additional challenges to be considered for proper interpretation of the results. Cancer cells are much more plastic than untransformed cells and are usually less differentiated or may even acquire characteristics of other cell lineages due to the expression of oncogenes and the deletion of tumor suppressor genes. This raises a possibility of untoward activation of gene promoters not normally active in a particular cell type. For example, abnormal activation of *Cre* expression may in turn lead to the confusion regarding the identity of cells from which cancer originates [5]. When considering liver cancer specifically, *Yap* oncogene was reported to be a potent inducer of hepatocyte dedifferentiation to cells with progenitor characteristics [6]. Expression of mutant *p53* is another inducer of dedifferentiation in liver cancer, and presence of both active Yap and mutant *p53* in murine livers caused the development of tumors with characteristics of undifferentiated progenitor cells [7].

To make the issue of cancer cell of origin even more complicated, hepatocytes may transdifferentiate into cholangiocytes during liver injury which typically precedes hepatocarcinogenesis. This process was recreated in murine livers by activating the Notch pathway [8]. Interestingly, transdifferentiation is responsible for biliary tree formation from hepatocytes in a mouse model of Alagille syndrome, in which cholangiocyte development is impaired because of reduced Notch signaling [9]. To our knowledge, the transdifferentiation of hepatocellular carcinoma cells into cholangiocellular carcinoma cells has not yet been described, but is certainly possible, especially in the context of chronic liver injury. By analogy, a reverse process is also theoretically possible. Thus, transdifferentiation implies changing a gene expression pattern characteristic of one cell type, for gene expression pattern characteristic of another cell type, and is likely preceded by global demethylation of mainly gene regulatory sites which facilitates the whole process [10].

Yet another phenomenon frequently occurring in cancer is epithelial-mesenchymal transition (EMT), in which epithelial cells lose their epithelial characteristics (e.g., epithelial markers expression), gain mesenchymal traits (e.g., mesenchymal markers expression), and acquire a more fibroblastic morphology and enhanced migratory properties. NOTCH and TGF-β pathways were implicated in inducing EMT, and in generation of cholangiocarcinomas from hepatocytes [5]. Moreover, EMT has been shown to contribute to both dedifferentiation and transdifferentiation, and is often induced in chronically damaged tissues, for example, cirrhotic liver, and was suggested to generate fibroblasts from hepatocytes in transgenic mice [5].

In summary, although genetically modified mouse models that rely on the Cre-LoxP system for conditional genetic modifications are powerful tools for interrogating gene functions in living organism, it poses caveats that require careful consideration. One limitation, the focus of this editorial, is the potential loss of fidelity of Cre recombinase expression especially in the context of modelling cancer in mice. Importantly, even a temporal induction of Cre recombinase expression in unintended cell types (due to dedifferentiation, transdifferentiation, EMT, and possibly other processes) may lead to deletion of *loxP* sites, a genetic change heritable by their progeny, which may be wrongly attributed to the effect of genetic modifications in the intended cell types, in effect potentially leading to erroneous conclusions.
